# The earliest segmental sternum in a Permian synapsid and its implications for the evolution of mammalian locomotion and ventilation

**DOI:** 10.1038/s41598-022-17492-6

**Published:** 2022-08-05

**Authors:** Eva-Maria Bendel, Christian F. Kammerer, Zhe-Xi Luo, Roger M. H. Smith, Jörg Fröbisch

**Affiliations:** 1 Museum Für Naturkunde, Leibniz-Institut Für Evolutions- und Biodiversitätsforschung, Invalidenstraße 43, 10115 Berlin, Germany; 2 Institut Für Biologie, Humboldt-Universität zu Berlin, Invalidenstraße 42, 10115 Berlin, Germany; 3 North Carolina Museum of Natural Sciences, 11 W Jones Street, Raleigh, NC USA; 4 Evolutionary Studies Institute, University of the Witwatersrand, Yale Road, Johannesburg, 2000 South Africa; 5 Department of Organismal Biology and Anatomy, University of Chicago, 1027 E 57th Street, Chicago, IL USA; 6 Department of Karoo Palaeontology, Iziko South African Museum, 25 Queen Victoria Street, Cape Town, 8001 South Africa

**Keywords:** Evolution, Palaeontology

## Abstract

The sternum is a stabilizing element in the axial skeleton of most tetrapods, closely linked with the function of the pectoral girdle of the appendicular skeleton. Modern mammals have a distinctive sternum characterized by multiple ossified segments, the origins of which are poorly understood. Although the evolution of the pectoral girdle has been extensively studied in early members of the mammalian total group (Synapsida), only limited data exist for the sternum. Ancestrally, synapsids exhibit a single sternal element and previously the earliest report of a segmental sternum in non-mammalian synapsids was in the Middle Triassic cynodont *Diademodon tetragonus*. Here, we describe the well-preserved sternum of a gorgonopsian, a group of sabre-toothed synapsids from the Permian. It represents an ossified, multipartite element resembling the mammalian condition. This discovery pulls back the origin of the distinctive “mammalian” sternum to the base of Theriodontia, significantly extending the temporal range of this morphology. Through a review of sternal morphology across Synapsida, we reconstruct the evolutionary history of this structure. Furthermore, we explore its role in the evolution of mammalian posture, gait, and ventilation through progressive regionalization of the postcranium as well as the posteriorization of musculature associated with mammalian breathing.

## Introduction

In the skeleton of tetrapods, the sternum acts as a ventral stabilizing element. It usually comprises a bony rod or plate in cartilaginous contact with the distal ends of the ribs, serving to reinforce the rib cage. It is also functionally associated with the pectoral girdle (part of the appendicular skeleton), which in Permian synapsids consists of the paired scapulae, procoracoids, coracoids (sometimes called metacoracoids^1^), cleithra, clavicles, and the unpaired interclavicle^2^. By anatomical convention, the sternum is not included in the pectoral complex, instead being classified as part of the axial skeleton, even though the ontogeny of these elements is interconnected^1^. Because of its functional importance, the pectoral girdle has been extensively studied in many fossil tetrapods. An animal’s stance and musculature can be inferred from the morphology of the pectoral girdle e.g.^3–6^, providing insights into its function and lifestyle. This is especially important in extinct clades, where one is generally not able to infer behavior by reference to extant representatives of the group. Despite its relevance to the function of the pectoral girdle in extant taxa, the sternum has received comparably little attention in fossil tetrapods. This is due in large part to its rarity of preservation; it is frequently lost in disarticulated specimens, and if it was cartilaginous in life (as in many extant reptiles and amphibians), it would be unlikely to fossilize.

In extant mammals the sternum is a well-ossified and segmental abaxial structure consisting of a large anterior element (the manubrium), a series of posterior segments (the sternebrae), and a usually pointed terminal (xiphoid) element, although in some cases these segments can fuse during ontogeny (e.g. in *Homo*). The sternal segments are also a prevalent and phylogenetically conserved pattern for all major Mesozoic clades of crown Mammalia e.g.^7–11^, with a single exception of *Zhangheotherium quinquecuspedens*^11^. The majority of extant reptiles also possess a segmental sternum, generally divided into (from anterior to posterior) pre-, meso-, and xiphisternum^12^. However, the homology of the sternal divisions of extant reptiles with those of mammals is questionable. Reptilian sterna are mostly cartilaginous in structure^2^ (with notable exceptions in Aves, in which the sternum forms an ossified keel-like structure for the attachment of flight muscles, and some lizards, e.g. *Iguana*^12^) and some reptiles have no sternum at all (i.e. snakes^13^). Hence, the fossil record provides only limited information on sternal evolution in reptiles, as the sternal cartilage rarely fossilizes.

**Figure 1:**
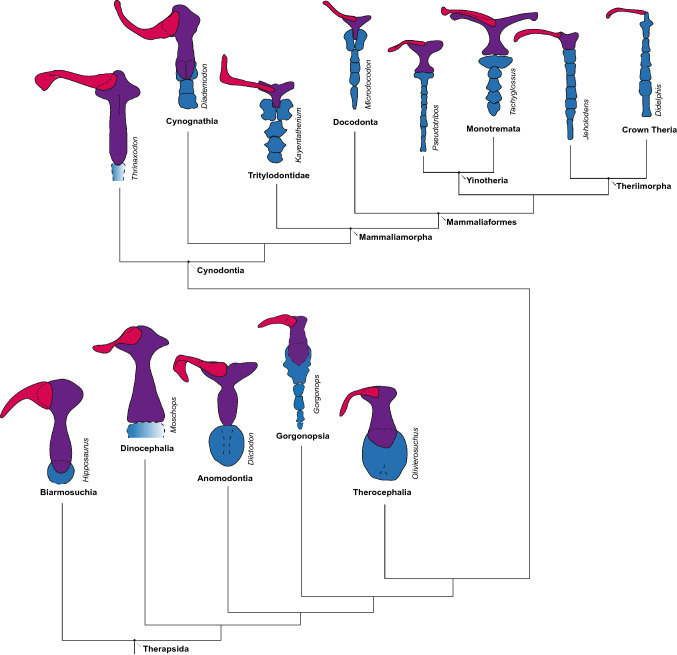
Schematic representation after Luo et al.^10^ of the evolution of the pectoral girdle elements interclavicle (purple), clavicle (pink) and sternum (blue) in the major therapsids clades Biarmosuchia (*Hipposaurus*^26^), Dinocephalia (*Moschops*^29^), Anomodontia (*Diictodon*^20^), Gorgonopsia (SAM-PK-K10591, *Gorgonops torvus*), Therocephalia (*Olivierosuchus*^21^) and the cynodont *Thrinaxodon*^71^, as well as Cynognathia (*Diademodon*^14^), Tritylodontidae (*Kayentatherium*), Docodonta (*Microdocodon*^9^), Yinotheria (*Pseudotribos*^10^ and the monotreme *Tachyglossus* (drawing after specimen ZMB-35995 in the Museum für Naturkunde, Berlin) and Theriimorpha (*Jeholodens*^10^ and the crown therian *Didelphis*^10^). All views from ventral. Simplified phylogeny after Hopson and Barghusen^24^ and Sidor and Hopson^25^.

However, the rich fossil record of late Paleozoic and Mesozoic taxa on the mammalian stem suggests that the modern mammalian sternum did not evolve from an ancestral morphology similar to that of extant reptiles^1^. No sternum is known in the earliest diverging synapsids (“pelycosaurs”), but the sternum is preserved in several non-mammalian therapsid groups and typically forms a single, large, rounded plate (Fig. 1). Previously, the earliest record of a mammal-like ossified segmental sternum was in the Middle Triassic *Diademodon tetragonus*^14^, a member of Cynodontia, the synapsid subclade that includes mammals. Based on this specimen, it had been assumed that the “mammalian-type” sternum was an apomorphy of cynodonts, one of the many mammalian characters that first appeared in this clade during their rapid radiation following the end-Permian mass extinction^15^.

Here, we present a mammal-like sternum in a late Permian gorgonopsian therapsid, which is phylogenetically stemward and geologically much earlier than the cynodont *Diademodon*. This specimen, referable to the species *Gorgonops torvus*, helps illuminate the transition between a primitive unipartite sternum and a derived multipartite (segmental) sternum along the mammalian stem-lineage. Placing the new specimen in the broader context of synapsid sternal evolution reveals a complex history of variation that provides insights into the functional diversity of early synapsids, and links it to the evolution of the mammalian thorax which was essential for the development for aspiration during breathing.

## Results

**Figure 2:**
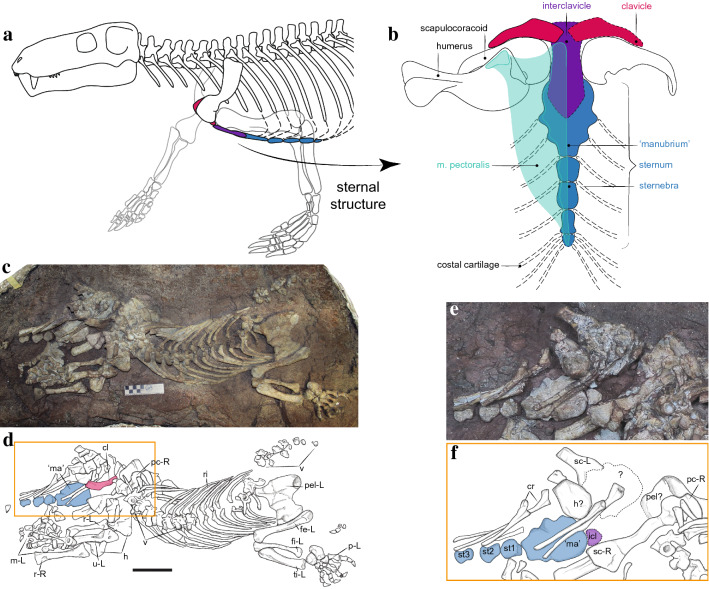
(**a**) Partial skeletal reconstruction of *Gorgonops torvus* in lateral aspect and (**b**) schematic reconstruction of the pectoral girdle, sternum, interclavicle and humerus with hypothetical position of m. pectoralis in ventral aspect. (**c**) Photograph and (**d**) interpretative drawing of *Gorgonops torvus* (SAM-PK-K10591), highlighting the pectoral region in an orange rectangle as an (**e**) photograph and (**f**) interpretative drawing (e and f after further preparation to reveal the interclavicle). For explanation of the taphonomic disturbance of the specimen, see Materials and methods section. Abbreviations: cl, clavicle; cr, cervical ribs; cv, cervical vertebrae; fe-L, left femur; fi-L, left fibula; h, partial humeri; icl, interclavicle; m-L, left manus; ’ma’, ‘manubrium’; p-L, left pes; pc-R, right pectoral girdle; pel?, partial pelvic girdle?; pel-L, left pelvic girdle; r-L, left radius; r-R, right radius; ri, ribs; sc-L; left scapula; sc-R, right scapula; st1-3, sternebra 1–3; ti-L, left tibia; u-L, left ulna; v, vertebrae.

The gorgonopsian specimen SAM-PK-K10591 (here referred to *Gorgonops torvus*, a mid-sized gorgonopsian recovered as an early-diverging member of the group in the most recent phylogenetic analysis^16^), constitutes a nearly complete skeleton. Of exceptional significance is the preservation in partial articulation of the pectoral girdle and associated sternum (Fig. 2). The sternum (visible dorsally) consists of a large, plate-like, roughly oval element and three smaller, flat elements. In other groups of therapsids with multipartite sterna, i.e., non-mammalian cynodonts, the former element is often called the ‘manubrium’ in anatomical literature^14,17,18^, as this is the term for the anteriormost and largest element of the sternum in Mammalia. But given the uncertainty regarding the homology between these elements^15^ we refer to this structure in quotes. In *Gorgonops torvus*, the ‘manubrium’ element bears two triangular articular facets for ribs on each side and shares a third articulation area with the first sternebra. The three smaller, discrete penta- or hexagonal elements, the sternebrae, are situated posterior to the ‘manubrium’. Just as the first sternebra contributes to the third rib attachment facet with the ‘manubrium’, additional ribs would have articulated lateral to the contacts between subsequent adjacent sternebrae via cartilage. This configuration of the sternum is similar to that of modern Mammalia, but contrasts with that of contemporary non-mammalian synapsids such as anomodonts e.g.^19,20^ and therocephalians e.g.^21,22^. In these groups, the ossified sternum commonly consists of a single large, rounded element and is generally considered unipartite.

Although damaged, part of the interclavicle is preserved as a plate-like element at the anterior edge of the sternum. Its posterior border is smaller than the anterior border of the ‘manubrium’ but is in close contact with it. The overlap of ‘manubrium’ and interclavicle is not preserved, but can be reconstructed based on other well-preserved gorgonopsian specimens (e.g. NHCC LB350, SAM-PK-9344, USNM 412381—see Supplementary Fig. S1). The right clavicle is a robust element preserved in dorsal view, which is slightly displaced from life position but shows a facet for articulation with the interclavicle, permitting reconstruction of the connection between these bones.

## Discussion

### Sternal morphology in Synapsida

The earliest-diverging synapsids, the paraphyletic “pelycosaurs”, do not preserve an ossified sternum in any known taxa^23^. However, a large, ossified interclavicle is always present. The broad interclavicle tends to be mostly uniform in shape (“spoon-shaped” as per Romer and Price^23^, with a cruciate anterior part and an elongate posterior rod). The first appearance of an ossified sternum in Synapsida occurs within the diverse and long-lived subclade Therapsida. Although some uncertainty exists as to the relationships between the major therapsid clades, the earliest-diverging group is generally considered to be Biarmosuchia^24,25^. Few biarmosuchian postcrania are known, but the sternum is preserved in a few taxa (e.g. *Hipposaurus*^26^), where it is unipartite and probably incompletely ossified. In known examples the sternum is relatively small compared to the interclavicle and roughly circular in outline (see Fig. 1). No sternum is known in the Dinocephalia^2,27^. As several nearly complete dinocephalian skeletons are known e.g.^28,29^, it seems that the sternum, if present, must have been cartilaginous in this group, and the lack of discovered sterna is not simply due to incomplete preservation of the bony elements (likely also the case for “pelycosaurs”).

Anomodontia is the most diverse Permo-Triassic therapsid clade^30^, and also exhibits a diversity of sternal morphologies. Although an ossified sternum seems to be lacking in basal (non-dicynodont) anomodonts, as indicated by its absence in the well-preserved and fairly complete skeletons of *Suminia*^31^, *Galechirus*, and *Galepus*^32^, an ossified sternum is present in Dicynodontia^30^. In dicynodonts, it is always unipartite and generally a simple, plate-like element (e.g. in *Diictodon*^20^ and *Eosimops*^33^). However, the sternum is more complex in the burrowing dicynodont *Cistecephalus* (wide anteriorly, with a strongly tapering posterior edge and pronounced attachment sites for the ribs)^19^. In the largest known dicynodonts, the Late Triassic stahleckeriids, the sternum is extremely deep dorsoventrally, with a well-developed ventral keel^6^. The number of ribs attaching to the sternum varies in the clade, with one (e.g. *Dinodontosaurus*^34^), two (e.g. *Aulacephalodon*^35^), or three (e.g. *Cistecephalus*^19^) attachment sites per side.

Few well-described postcrania are known for Gorgonopsia. Previously-described gorgonopsian sterna consist of one element with up to three articulations for ribs on either side (i.e. in the holotypes of *Lycaenops ornatus*^36^, *Aelurognathus tigriceps*^36^, “*Aelurognathus*” *microdon*^37^, and *Viatkogorgon ivakhnenkoi*^38^). The discovery of an ossified and segmental abaxial sternal structure in *Gorgonops torvus*, however, raises the possibility that the apparently unipartite sterna of other species reflect incompleteness rather than the true absence of discrete sternebrae. With the exception of *V*. *ivakhnenkoi*, the aforementioned specimens were all collected and prepared in the early twentieth century, with damage to the more delicate parts of the anatomy. Also, although complete, well-preserved, and well-prepared, the skeleton of *V*. *ivakhnenkoi* is preserved on its side, and the base of the pectoral complex is poorly exposed, making the morphology of the sternum somewhat uncertain.

Similar to the condition in Gorgonopsia, few skeletons of Therocephalia are complete enough to determine whether a sternum was present. An ossified sternum appears to be absent in basal (non-eutherocephalian) therocephalians, as no trace of this element is present even in well-preserved, articulated skeletons of this grade (i.e. *Glanosuchus*^39^, *Lycosuchus*^40^). However, an ossified sternum is known in a number of eutherocephalian taxa (e.g. *Regisaurus*^22^ and *Olivierosuchus*^21^) and likely was present throughout that subclade^41^. In these taxa, the preserved portion of the sternum consists of a single element and is a remarkably large, plate-like structure dwarfing the interclavicle (Fig. 1).

Prior to the discovery of the gorgonopsian specimen described here, the earliest record of an ossified multipartite sternum was in the Middle Triassic cynodont *Diademodon tetragonus*^14^. No ossified sternal elements are known in any earlier cynodonts (including taxa known from numerous complete skeletons, such as *Thrinaxodon*), suggesting that the sternum was cartilaginous in those taxa. Therefore, no conclusions can be drawn about the sternal shape in the earliest cynodonts. However, a multipartite sternum is known in several later-occurring non-mammalian cynodonts (e.g. the Jurassic *Kayentatherium wellesi*^17^ and *Bienotheroides wansienensis*^18^), in which the anteriormost section of the sternum is paired. Although rare, all the non-mammalian cynodont sterna thus far described consist of multiple elements. The connection between all these elements is assumed to have been cartilaginous^18^.

A fully-ossified multipartite sternum is known in several extinct mammaliaform taxa (e.g. *Sinoconodon*^42^, *Maiopatagium*^43^, *Microdocodon*^9^) (Fig. 1) as well as all modern mammals^44^. Adult monotremes and non-crown group therians retain a distinct interclavicle, which acts as an anchor for the proximal attachment of the clavicle, and the first rib attaches to the largest anterior sternal element (the manubrium). Marsupials and placentals do not preserve an interclavicle as adults, as this element fuses with the manubrium during development. In these taxa, the clavicles and the first ribs both connect to the anteriormost sternal element on either side^45^.

The new multipartite sternum of a gorgonopsian presented here appears substantially earlier in geological time and is phylogenetically more stemward than any previous records of a “mammalian-type” sternum. The partial interclavicle shows some similarities to the interclavicles in other gorgonopsian specimens (see Supplementary Fig. S1) as well as those of Therocephalia (e.g. *Olivierosuchus*^21^), but the sternum of *Gorgonops torvus* is novel in its configuration.

The sternal variation within Synapsida discussed above allows us to distinguish between three morphologically differentiated groups:

A) Synapsids inferred to have an unossified sternum, such as “pelycosaurs”, dinocephalians, and basal anomodonts, therocephalians, and cynodonts. The lack of an ossified sternum in the predominantly large-bodied Dinocephalia demonstrates that sternal ossification is not necessarily correlated with body size.
B) Synapsids with usually large, unipartite (singular), and well-ossified sterna, for instance dicynodonts and eutherocephalians. Although it is possible that additional cartilaginous elements were present in life, the lack of a well-developed articular facet on the posterior margin of the sternum in these groups suggests that is unlikely.
C) Synapsids with segmental and ossified sterna such as *Gorgonops torvus*, *Diademodon tetragonus,* Mesozoic mammaliaforms*,* and extant mammals. The condition in close relatives of *Gorgonops* and *Diademodon* is uncertain, due to limited fossil data.

The discovery of the sternal complex of *Gorgonops torvus* now presents two equally possible hypotheses for the earliest evolution of the mammalian sternum: 1) the “mammal-like” condition arose first in gorgonopsians (as represented by *Gorgonops torvus*) but then was lost in eutheriodonts (therocephalians and cynodonts, in which the sternum ancestrally seems to have been cartilaginous) or 2) the condition in *Gorgonops torvus* evolved convergently to that of cynodonts, originating from a unipartite ancestral state common to both gorgonopsians and eutheriodonts. Until further discoveries of fossil taxa with different sternal conditions provide more evidence, it is impossible to test either of these hypotheses thoroughly, but functional considerations may provide some insight as to which is more likely (see below).

### Functional evolution of the sternum

The sternum of extant mammals has several functions. Notably, it helps to reinforce the rib cage, with a more stable, enclosed rib cage offering better protection of the thoracic organs than one exposed abaxially^46^. Furthermore, an ossified (and hence stronger) sternum is functionally important for forelimb locomotor function, as the ventral surface of the thorax has major attachment sites for pectoral muscles^47^. These complementary functions of the sternum reflect its integral part in the entire system of the forelimb, the shoulder girdle, and the thorax. In synapsid evolution, there are two major morphologies of ossified sterna (Fig. 1): the single, plate-like sternum present in earlier-diverging synapsids (e.g. dicynodonts) and the relatively narrow, segmental sternum seen in cynodonts such as *Diademodon*, some tritylodontids and mammaliaforms. The shift between these osteological configurations would have been part of a broader suite of functional changes occurring in this section of the synapsid tree.

The origin of mammals is associated with major changes in skeletal morphology, and the stepwise assembly of these changes in Permo-Triassic synapsids has historically been cited as one of the best bodies of evidence for macroevolution in the fossil record^48,49^. The inferred functional associations (and evolutionary drivers) of these changes can be roughly broken down into three areas: 1. dental (increasing complexity, both from differentiation in the heterodont tooth series, and from elaboration of individual teeth, particularly the postcanines, with multicusped and expanded crowns capable of occlusion); 2. cranial (formation of a complete secondary palate, loss of the postorbital bar, simplification of the jaw elements, increase in brain size/complexity); and 3. postcranial (increased regionalization of the axial column, changes in limb morphology associated with posture, origin of the segmental sternum). Each of these changes has functional implications—more efficient food processing driven by changes to the inferred muscular complement and jaw orientation for the craniodental characters^50^, and more active locomotion associated with an erect gait for the postcranial characters^51^. Each of these had downstream effects on portions of the anatomy not immediately subject to selection. For example, the expansion of jaw musculature attachment on the dentary is thought to have contributed to the decrease in size of the post-dentary bones and their eventual detachment to form middle ear bones^52^.

We offer a similar interpretation for the evolution of a segmental sternum in Permo-Triassic therapsids. On its own, this feature would have had little to do with improved gait in mammals—the forelimbs themselves, the shoulder girdle, and the thoracic vertebral column all have more immediate influences on locomotion. However, the sternum bridges the girdle to the axial skeleton and it is therefore connected with shifts in locomotor evolution. And it is involved in two ways of particular note in the evolution of mammal-like morphologies and function: 1. increased regionalization of the axial skeleton and 2. increased posteriorization of thoracic elements. For the former, mammals are well known to have greater differentiation of the axial column into discrete regions than reptiles, although this transition is now thought to be more complex and to have occurred earlier in synapsid evolution than previously believed^53^. In the typical mammalian condition, the thorax is a highly discrete unit readily distinguished by vertebral morphology, and it also differs in range of motion from the cervical, lumbar, and caudal regions. By contrast, in many reptiles and even early synapsids, the distinction between the thoracic and lumbar regions is less evident, and the cervical-thoracic transition is also difficult to discern^54^. The origins of the mammal-like rib cage, a structure surrounding the thoracic organs (the heart, lungs and muscular diaphragm), are intimately associated with changes in gait that took synapsids from the lateral undulation of early amniotes to the primarily dorsoventral flexion of mammals^47^, in a divergent evolutionary path from the evolution of modern reptiles^55^. In the context of this paradigm shift in synapsid history, a massive, plate-like sternum broadly overlapping the interclavicle would have been a hindrance, a relic of the “pelycosaurian” condition with sprawling forelimbs in close association with the substrate. In the evolution of theriodonts (the group containing gorgonopsians, therocephalians, and cynodonts), even as early as gorgonopsians there is a shift towards more cursorial locomotion and more erect gaits, with a focus on dorsoventral rather than side-to-side motion^51,55^. To facilitate this style of locomotion, it was necessary to reduce the size of the pectoral girdle, thereby enhancing its mobility relative to the axial skeleton.

There are multiple ways to reduce the weight of bony elements, one being simply to not ossify them. This may have been the ancestral condition in eutheriodonts, given that the sternum seems to have been cartilaginous in the earliest therocephalians and cynodonts (although this would imply a reversal to the pre-theriodont condition in eutherocephalians). Another is to transform from a single solid plate to a series of connected elements, which can retain the protective function of the sternum without limiting mobility (similar transitions can be seen in the evolution of armor, with trends towards multipartite structures offering greater flexibility^56^). This latter approach appears to characterize sternal evolution in Gorgonopsia.

Greater flexibility of the thorax also has importance beyond permitting dorsoventral flexion during locomotion, as shown by Jones et al.^53,55^ in their studies of the axial skeletal evolution in Synapsida. Increased potential for axial twisting can also aid in behaviors such as grooming and fast locomotory maneuvers, but this requires vertebral specializations for torsion. In earlier non-mammalian synapsids (i.e. most non-cynodont taxa), the functional regions of the vertebral column are not as distinct as in later taxa such as advanced cynodonts (e.g. the Jurassic *Kayentatherium*^55^), and there is little evidence of selection for performance under torsion in the anterior vertebrae. However, a general phylogenetic trend towards more regionalization into pre- and post-diaphragmic areas of the vertebrate column can be observed even in more stemward portions of synapsid phylogeny^55^. A more flexible, segmental sternum, as seen in *Gorgonops torvus*, may represent a prerequisite for accommodating intervertebral torsion in the thorax.

Therefore, we hypothesize that the evolution of the ossified segmental sternum in Theriodontia is a part of the broad evolutionary shift towards more mammal-like locomotion, which may have facilitated the rise of this group as the dominant carnivores of the late Permian. Selection for a lighter, more flexible sternum in the context of changing posture, gait, and vertebral mobility can be inferred regardless of the homology of the segmental sternum in *Gorgonops*—either this morphology evolved convergently in gorgonopsians and eucynodonts, or it would represent an ancestral adoption retained in cynodont evolution (albeit cartilaginous in taxa other than eucynodonts).

However, posture and gait were not the only major changes in thoracic anatomy occurring in Permo-Triassic therapsids. The transition to a mammal-like thoracic morphology is also tied to the way for therapsids to break Carrier’s constraint: the respiratory limitation driven by dual use of the axial musculature during lateral flexion and costal breathing during rapid locomotion^47^. Dorsoventral flexion in mammals, and a more rigid thorax centered more anteriorly along the vertebral column, fundamentally altered synapsid ventilation, permitting both lungs to be expanded or compressed simultaneously, a metabolically more efficient method advantageous for active locomotion. For this to work, however, it is necessary that the dorsal and ventral limits (i.e. the vertebral column and sternum) of the bony enclosures of the lungs (i.e. the rib cage) are both strong and pliable, conferring functional advantage over a single stiff interclavicle-sternal plate in managing volume of the thoracic cavity^57^. A multipartite sternum with cartilaginous tissue between the ‘manubrium’ and the sternebrae is consistent with this requirement. However, while this on its own would have helped to reduce the impact of Carrier’s constraint, actually breaking the constraint required an additional innovation: the diaphragm, a muscular sheet at the base of the thoracic cavity capable of pumping air through the lungs independently of locomotion.

Amongst the basic requirements for a diaphragm is that it must functionally be positioned caudad to the sternum, because by contracting during respiration, it creates negative pressure in the chest that is stabilized by the robust yet flexible complex of ribs, costal cartilages, and the segmental sternum. The origins of the diaphragm are obscure, however; it has been proposed to be unique to mammals or to have originated in some of the earliest “pelycosaurs” (e.g. caseids)^58^. Recent research taking data from developmental studies suggests that the diaphragm originated from ancestral pharygneal muscles of the cervico-thoracic region by posteriorization of elements associated with it, i.e. the forelimb bud during development and the brachial plexus nerve^59^. Accordingly, if the diaphragm did indeed originate from cervico-thoracic pharyngeal muscles, then the two requisite changes associated with the diaphragm may have been well underway in gorgonopsians: a) the posteriorization, evidenced by the likely presence of seven cervical vertebrae^60^ and the herein described elongate segmental sternum. And b) the elongate configuration itself of the sternum of *Gorgonops,* providing the needed caudad-positioned attachment for the diaphragm. This indicates that a mammalian-style diaphragm should already have been present in this taxon (and possibly, by inference, in theriodonts generally) to support the changes in ventilatory function.

Ontogenetic development of the sternum is well studied in extant mammals, with a particularly robust literature in the realms of human medicine and mouse embryology, demonstrating that formation of the characteristic segmental sternum is mediated by interactions with the developing ribs^15,61^. Specifically, the rib tips inhibit skeletal maturation, resulting in ossification of the intermediary regions but maintenance of cartilaginous connections between them^62^. As such, we must consider whether the segmental sternum would even have been selected for at all, or merely is an inherent consequence of developmental formation of a thoracic rib cage between the axial skeleton and sternum. Here, the fossil record is instructive. The plate-like sternum of dicynodonts has a variable number of rib attachments (see above), but a number of taxa clearly show multiple ribs attached to the single sternal element^19^. Therefore, it is apparently not an inherent developmental feature of Synapsida that rib attachments inhibit sternal growth and cause segments of the sternebrae to form. Rather, we propose that this system evolved through co-opting developmental mechanisms during a period of selection towards lighter and more jointed thoracic structures. Unfortunately, the cartilaginous nature of these elements in many synapsid groups (notably early cynodonts) makes it difficult to establish a precise understanding of the shift between dicynodont- and therocephalian-like structures and those of mammals. However, discoveries like that of the new *Gorgonops* specimen provide strong support for an early origin of the functional suite of derived mammalian locomotion and ventilation in the Permian antecedents of the clade.

## Materials and methods

The gorgonopsian specimen SAM-PK-K10591 was discovered, excavated and mechanically prepared by Georgina Farrell (see Fig. 2c), and more recently given additional preparation by Nyaniso Nofingxana (see Fig. 2e) at the Iziko South African Museum in Cape Town. The skeleton is almost complete and maintained in situ on the slab to retain preservational context. SAM-PK-K10591 was found on the farm Wilgersbosch Kloof 449 in Oukloof Pass (Lat -32.187473 Long. 21.820334), some 120 km south east of Fraserburg in the Northern Cape Province, South Africa. The locality is in a low sandstone-capped cliff exposure of *Tropidostoma*-*Gorgonops* Subzone^63,64^ mudrocks, indicating an early late Permian (Wuchiapingian) age. A partial cranium was found as several loose pieces in close association with the semi-articulated postcranial skeleton, allowing for confident identification of the specimen as *Gorgonops torvus* (i.e., the presence of four postcanines, a prominent precanine ‘step’ to the maxillary alveolary margin, and proportions of the snout are diagnostic—for measurements of the skull, see Supplemementary Table S1).

The thinly bedded mudrocks that host the specimen contain isolated smooth surfaced calcareous nodules and claystone-lined root casts indicative of an immature palaeosol in a distal floodplain paleoenvironment^65^. The bones are patchily peri-mineralized with a thin, 5 mm-thick encrustation of the same micritic nodular material. Such bacterially mediated precipitation of carbonate suggests that most of the skeleton was buried before the soft tissue had completely decomposed^66,67^. The presence of fine longitudinal cracks in the limb bones represents Behrensmeyer’s^68^ weathering stage 1 and is an indication that the bones were exposed to the elements for 0.5 to 2.5 years before final burial.

The unusual distribution of elements of the SAM PK-K10591 skeleton is worthy of further description, as it indicates what happened in the post-mortem/pre-burial phase. When discovered, only the anterior part of the skeleton was partly exposed, the posterior half being fully enclosed in mudrock. Thus, the five articulated dorsal vertebrae that have been removed from the carcass (see Fig. 2c and d), without displacing the articulating ribs, must have occurred before burial. This atypical occurrence, combined with an unnatural 180-degree rotation of the neck (and likely the skull as well, although this cannot be confirmed) along with its articulated cervical ribs (see Fig. 2) and complete sternum, is interpreted as most likely the work of scavenging tetrapods. Efforts by co-occurring carnivores (likely other therapsids) to dismember the carcass may have been hampered by tough desiccated skin and connective tissue, which not only resisted their attempts, but also kept the ribs and sternum in articulation. Similar taphonomic modification is observed in modern mid-sized carcasses that have been scavenged by non-bone-cracking predator/scavengers such as jackals and coyotes, e.g.^69,70^.

## Supplementary Information

Supplementary Information.

## Data Availability

All data generated or analyzed during this study are included in this published article.
