# Supplementary material for: The earliest segmental sternum in a Permian synapsid and its implications for the evolution of mammalian locomotion and ventilation
Source: Sci Rep. 2022 Aug 5;12:13472. doi: 10.1038/s41598-022-17492-6 (PMC9356055; doi:10.1038/s41598-022-17492-6)
Supplement: Supplementary file 1 — Supplementary Information. [file 41598_2022_17492_MOESM1_ESM.pdf]

The earliest segmental sternum in a Permian synapsid and its implications for the evolution of mammalian locomotion and ventilation

Eva-Maria Bendel*[1,2], Christian F. Kammerer[3,4], Zhe-Xi Luo[5], Roger M.H. Smith[4,6], Jörg Fröbisch[1,2,4]

[1]Museum für Naturkunde, Leibniz-Institut für Evolutions- und Biodiversitätsforschung, Invalidenstraße 43, 10115 Berlin, Germany
[2]Institut für Biologie, Humboldt-Universität zu Berlin, Invalidenstraße 42, 10115 Berlin, Germany
[3]North Carolina Museum of Natural Sciences, 11 W Jones Street, Raleigh, NC, USA
[4]Evolutionary Studies Institute, University of the Witwatersrand, Yale Road, Johannesburg, 2000, South Africa
[5]Department of Organismal Biology and Anatomy, University of Chicago, 1027 E 57th Street, Chicago, IL, USA
[6]Department of Karoo Palaeontology, Iziko South African Museum, 25 Queen Victoria Street, Cape Town, 8001, South Africa
* Eva-Maria Bendel.

Supplementary material.

Supplementary Table 1.
Basic measurements of skull of *Gorgonops torvus* (specimen SAM-PK K10591)

Skull length (estimated):	ca. 30 cm
Snout length:	ca. 13 cm
Height of snout:	ca. 10 cm (measured from ventral most part of maxilla to skull roof)
Skull width:	ca. 6.5 cm (although extremely laterally compressed)

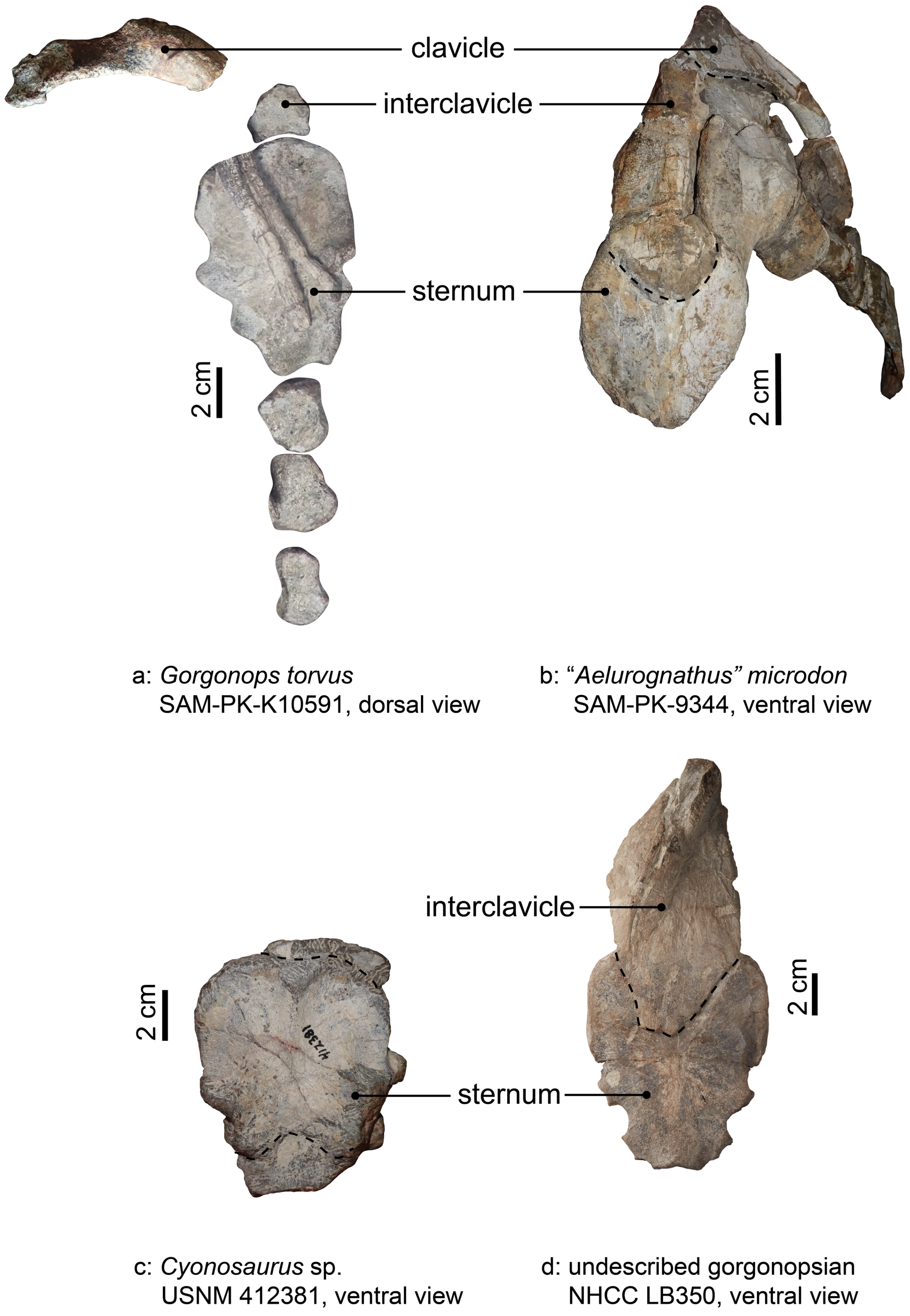

Supplementary Figure 1. Gorgonopsians preserving the sternum and/or interclavicle, used as a base for the reconstruction in Fig. 2b. Photograph in SFig 1d adapted from Christian A. Sidor, all rights reserved.
